# Poly-crisis and global health: how can we improve human health and equity while protecting the planet? The 30th Canadian Conference on Global Health in 2024

**DOI:** 10.7189/jogh.15.02001

**Published:** 2025-03-14

**Authors:** Michelle Amri, Margaret J Mutumba-Nakalembe, Johanna C Manga, Colleen M Davison, Fawad Akbari

**Affiliations:** 1 The W. Maurice Young Centre for Applied Ethics, School of Population and Public Health, University of British Columbia, Vancouver, British Columbia, Canada; 2 Frugal Biomedical Innovations, School of Biomedical Engineering, Western University, London, Ontario, Canada; 3 Canadian Association for Global Health, Ottawa, Ontario, Canada; 4 Department of Public Health Sciences, Queen's University, Kingston, Ontario, Canada; 5 Grand Challenges Canada, Toronto, Ontario, Canada

The Canadian Association for Global Health convenes the Canadian Conference on Global Health (CCGH) annually to provide a national platform to discuss pertinent global health issues. The 30th Canadian Conference on Global Health, co-chaired by Dr Michelle Amri and Dr Margaret Mutumba, was held in person from 25 to 27 October 2024 in Vancouver and virtually [1]. The CCGH 2024 brought together over 700 registered delegates from across 52 countries to discuss the conference theme, which was ‘poly-crisis and global health: how can we improve human health and equity while protecting the planet?’.

Despite remarkable and unprecedented gains in global health from 1990 to 2015, including the adoption of the 17 Sustainable Development Goals by 193 United Nations Member States in 2015 [2], the world has experienced various shocks in recent years. Namely, a series of economic, social, geopolitical, and environmental shocks have impacted our development, well-being, and collective future. The COVID-19 pandemic taught us that inadequately addressed preexisting problems exacerbate crises and make policy responses more challenging [3]. In Canada, we have seen inequities exacerbated by concomitant crises affecting health, education, housing, and ecological systems. Globally, increasing state-sponsored violence over the past three years, such as in Artsakh, Ethiopia, Gaza, Sudan, and Ukraine has also had various impacts. The World Bank Group has estimated that by 2030, nearly 60% of the world’s ‘extreme poor’ will live in countries affected by fragility, conflict, and violence [4]. This combination of events, the COVID-19 pandemic, extreme climatic events, degradation of food and land systems, global instability precipitated by war and conflict, high energy prices, and inflation, have stalled and even reversed progress. This has been termed ‘poly-crisis’, meaning that there has been a ‘convergence of the COVID-19 pandemic, escalating global conflicts, economic instability and climate change (…). These crises interact and amplify each other’s impact, disproportionately affecting vulnerable populations and exacerbating poverty and inequalities globally’ [5]. In a more simplistic sense, we understand this term to mean an interconnected web of simultaneous adversities manifesting interdependently in a globalised world. Through complex and interrelated pathways, these critical events impact our health, well-being, and planet.

The CCGH 2024 brought together researchers, practitioners, policymakers, advocates, students, and others within the global health community, as well as from other fields and those with lived experiences, to exchange learnings and ideas. Discussions bridged research and practice to better understand how this multifaceted challenge could be addressed to improve human and planetary health outcomes from a global public health perspective. Thus, the CCGH 2024 sought to address the drivers and immediate impacts of poly-crisis and lay the foundation for more equitable, inclusive, resilient, and sustainable global health ecosystems. Emphasis was placed on equity, social justice, community engagement, proactive involvement of populations in situations of vulnerability and marginalisation, and authentic partnerships between researchers and practitioners from the global south and global north. Under this broader theme, the CCGH 2024 featured two sub-themes, which are detailed below.

## SUB-THEME 1: UNPACKING THE DRIVERS OF POLY-CRISIS

This first sub-theme focussed on critically examining structural factors that drive the poly-crisis, such as politics, policy, governance, leadership, accountability, and power dynamics. We felt that we cannot act without a fulsome understanding of the drivers of poly-crisis. As such, this sub-theme sought to unpack drivers at various levels (*e.g.* global, national, subnational) and included discussions around political economy; governance, leadership, and accountability; and power dynamics, power structures, and empowerment. First, by discussing political economy, which ‘focuses on power and resources, how they are distributed and contested in different country and sector contexts, and the resulting implications for development outcomes’ [6], we were able to analyse how historical, political, and socioeconomic factors intersect and exacerbate health inequities and crises. Second, we delved into lessons learned and evidence-informed best practices around strengthening governance structures, accountability, and leadership mechanisms. We focussed on governance, leadership, and accountability as it can lead to ensuring responsive, transparent, inclusive, and equitable health systems. Third, we opened the floor to engaging conversations around distributing power and resources more equitably at global, national, and local levels. We emphasised pathways for community empowerment – particularly populations in situations of economic and political vulnerability or marginalisation – to enable the engagement of diverse perspectives in the development of public policy and programmatic interventions.

## SUB-THEME 2: MITIGATING AND ADAPTING TO THE IMPACTS OF POLY-CRISIS

This second sub-theme focussed on exploring innovative mechanisms and adaptive strategies to effectively reduce the impacts of simultaneous and intersecting crises. We expressed a commitment to evidence-informed interventions and enhancing resilience against poly-crisis. This sub-theme included discussions on evidence-informed policymaking; governance; innovation in health financing for health system strengthening; and technological innovations. First, we focussed on evidence-informed policymaking to ensure interventions are grounded in scientific evidence and community-based knowledge, thereby aiming to enhance effectiveness and sustainability. Second, we sought to promote and implement strategies to strengthen inclusive, equitable, and transparent governance mechanisms and systems. We emphasised the need for ‘good’ governance to promote accountability, be data-driven, engage in participatory decision-making, and have strong leadership. For instance, we welcomed discussions on intersectoral and multisectoral collaboration and undertaking health equity impact assessments. Third, we explored innovative health financing models, such as impact investing, blended finance, global health bonds, and fiscal and taxation systems. These have the potential to support efficient resource mobilisation to sustainably address crises at the national level with locally generated innovations. Lastly, we welcomed conversations that harness the power of artificial intelligence, big data, e-health, and m-health, while capitalising on existing local systems. These conversations also included digital surveillance systems that predict, monitor, and mitigate the effects of crises on health systems.

## CONCLUSION

Following the CCGH 2024, the Canadian Association for Global Health conducted an evaluation survey of all registrants, which included attendees, speakers, and sponsors. The survey highlighted that many of the 140 respondents felt that the conference increased their knowledge and/or awareness on global health research, policies, and best practices (83.9%); interest in their work (81.0%); professional networks (78.4%); and capacity to develop strategies for collaboration in addressing the effects of polycrisis on health and health inequities (74.4%).

We built on the successes of the CCGH 2023 [7] to host the CCGH 2024, which was an overwhelming success by many measures. However, we still grapple with one challenge: access to visas. Although we provided letters of support for suitable applicants to use in their visa applications, the decisions around visas and significant wait times required are outside of the control of the Canadian Association for Global Health. We are deeply upset that many of our global colleagues were unable to attend this event in-person because of visa issues and we recognise that this perpetuates inequities in the field. Ensuring the conference is hybrid was immensely important to us as a small step to engage colleagues who are interested in having these important conversations about global health. The CCGH has a strong history and track record of welcoming colleagues from around the world and we have strived at every step of the way to continue this legacy. As such, we invite the global health community globally to join us – whether in-person or virtually – for the next CCGH to be held in Halifax, Canada from 24 to 28 October 2025.

## References

[1] Canadian Association for Global Health. 2024 Canadian Conference on Global Health. 2024. Available: https://cagh-acsm.org/en/events/2024-canadian-conference-global-health-0. Accessed: 25 February 2025.

[2] Government of Canada. The 2030 Agenda for Sustainable Development. 14 June 2024. Available: https://www.international.gc.ca/world-monde/issues_development-enjeux_developpement/priorities-priorites/agenda-programme.aspx?lang=eng#. Accessed: 25 February 2025.

[3] Amri MM Drummond D Punctuating the equilibrium: an application of policy theory to COVID-19. Policy Des Pract. 2021;4:33–43.

[4] World Bank Group. Fragility, Conflict & Violence. 24 May 2024. Available: https://www.worldbank.org/en/topic/fragilityconflictviolence/overview#1. Accessed: 25 February 2025.

[5] Rasella D Macicame I Naheed A Naidoo M Landin-Basterra E Silva N The need for global social epidemiology in the polycrisis era. BMJ Glob Health. 2024;9:e015320. 10.1136/bmjgh-2024-015320 38642929 PMC11033662

[6] Reich MR Political economy analysis for health. Bull World Health Organ. 2019;97:514. 10.2471/BLT.19.238311 31384066 PMC6653823

[7] Amri M Mutumba-Nakalembe MJ Manga JC Davison CM ‘From rhetoric to action: Moving policy, research, and practice’ - The 29th Canadian Conference on Global Health in 2023. J Glob Health. 2024;14:02001. 10.7189/jogh.14.02001 39666582 PMC11636949

